# Metatranscriptomic and Metabolomic Insights Reveal Enhanced Colonial *Microcystis aeruginosa* Tolerance to Erythromycin in P-Limited Environment

**DOI:** 10.3390/toxics14080660

**Published:** 2026-07-27

**Authors:** Lei Jiang, Li-Jun Zhou, Shengxing Wang, Siwen Chen, Xiaoli Shi, Qinglong L. Wu, Kaining Chen

**Affiliations:** 1 State Key Laboratory of Lake and Watershed Science for Water Security, Nanjing Institute of Geography and Limnology, Chinese Academy of Sciences, Nanjing 211135, China; 2 University of Chinese Academy of Sciences, Beijing 100049, China; 3 School of Life Sciences, Institute of Life Science and Green Development, Hebei University, Baoding 071002, China; 4 Key Laboratory of Aquatic Ecosystem Health in the Middle and Lower Reaches of Yangtze River, Ministry of Ecology and Environment, Jiangsu Provincial Academy of Environmental Science, Nanjing 210036, China; 5 Key Laboratory of Integrated Regulation and Resource Development on Shallow Lakes, College of Environment, Hohai University, Nanjing 210098, China; 6 Center for Evolution and Conservation Biology, Southern Marine Sciences and Engineering Guangdong Laboratory (Guangzhou), Guangzhou 511458, China; 7 Sino-Danish Center for Science and Education, University of Chinese Academy of Sciences, Beijing 100039, China; 8 The Fuxianhu Station of Plateau Deep Lake Research, Chinese Academy of Sciences, Yuxi 653100, China; 9 Collaborative Innovation Center of Technology and Material of Water Treatment, Suzhou University of Science and Technology, Suzhou 215000, China

**Keywords:** erythromycin, colonial *Microcystis aeruginosa*, phosphorus deficiency, metatranscriptomic, metabolomic

## Abstract

As the primary component of harmful algal blooms, cyanobacteria exhibit unique adaptation strategies under environmental stress. The impact of erythromycin (ETM), a common macrolide antibiotic in aquatic environments, on colonial cyanobacteria remains unclear. This study examined the chronic toxic effects of different ETM concentrations (0.01, 0.1, 1, 10, 20 and 100 μg/L) on colonial *Microcystis aeruginosa* (*M. aeruginosa*) under varying nutrient conditions. Results showed that at 0.01–1 μg/L, ETM could promote the growth of *M. aeruginosa*, while high concentrations of ETM (≥10 μg/L) significantly inhibited growth (*p* < 0.05). Low-level ETM exposure accelerates *M. aeruginosa* growth by boosting PSII efficiency, extracellular polymeric substance (EPS) production, and the activities of superoxide dismutase (SOD) and catalase (CAT). Metatranscriptomic and metabolomic analyses further reveal that this stimulation is underpinned by enhanced trace-element uptake, reinforced carbon cycling, and increased biosynthesis of proteins, polysaccharides, and chlorophyll precursors. High-level ETM exposure inhibited the growth of *M. aeruginosa*, as evidenced by decreased photosynthesis, damaged membranes and suppressed metabolic activity. Metatranscriptomic and metabolomic analyses showed the upregulation of photosynthesis andpathways, metabolic pathways, and the accumulation of potent allelochemicals in P-limited cells exposed to 10 µg/L ETM relative to 10 µg/L ETM in nutrient-replete BG11 medium. Furthermore, phosphorus deficiency may enhance the potential of net methane formation. These findings underscore the complex interactions between antibiotic exposure and nutrient stress in cyanobacteria, with significant implications for environmental management of antibiotic contamination.

## 1. Introduction

Macrolide antibiotics (MLs) are now among the most frequently detected antimicrobial residues in aquatic systems and warrant urgent attention [1]. It was a commonly prescribed drug, second only to penicillin, in the United States [2]. In China, MLs constituted the largest share of the overall antibiotic consumption, especially in the livestock industry [3]. For example, in the Lijiang River surface water, the total concentration of MLs ranged from 42.47 to 1674.30 ng/L (mean 502.69 ng/L) [4]. In the Weihe River and its tributaries, macrolide antibiotics also reached notably high concentrations [5]. Erythromycin (ETM) is a widely used macrolide antibiotic in clinical practice. It consistently shows a higher detection rate in aquatic environments of China [6], Europe [7] and the United States [8] than other MLs. It has been reported that the average concentrations of ETM in surface water of the Jianghan Plain (central China) reached up to 1.60 μg/L in spring, 0.772 μg/L in summer, and 0.546 μg/L in winter [9].

Residual antibiotics in the environment could have effects on non-target organisms. Previous studies have demonstrated that algae are more sensitive to antibiotics than daphnia and fish [10,11]. Cyanobacteria, due to their higher sensitivity to antibiotics than eukaryotic algae, are considered valuable indicative species in the environmental risk assessment of antibiotics [12]. ETM has been proven to exert hormesis effects on the cell growth of *Microcystis*, with low concentrations promoting growth [13], while high concentrations reduce the quantum yield of PSII in *Microcystis aeruginosa* [14]. Additionally, exposure to high concentrations of erythromycin can cause damage to the DNA replication pathway in algae [15]. Most studies were performed by using unicellular cyanobacteria under laboratory culture conditions [16]. However, in natural environments, cyanobacterial cells often aggregate to form colonies, comprising multiple cells and the extracellular polymeric substances (EPS) they secrete [17]. Colonial cyanobacteria, such as *Microcystis*, are the main microorganisms that cause cyanobacterial blooms in eutrophic lakes [18]. Additionally, colonial cyanobacteria are often exposed to antibiotics for prolonged periods [19]. Cyanobacterial colonies are effective at resisting various environmental stresses in nature, including low-nutrient conditions, solar radiation [20], and predation [21]. It is worth further exploring whether the effects of antibiotics on the colonial cyanobacteria with size advantages are different [22].

Nutrient availability constrains the rate of biological production and ecosystem structure globally [23]. Though both nitrogen and phosphorus are vital for phytoplankton growth in freshwater systems [24], phosphorus loading is generally considered to be the main driver of lake eutrophication. As a core strategy for ecological restoration, external nutrient control has been extensively applied in the management of some eutrophic lakes [25], leading to significant reductions in total phosphorus (TP) concentrations and a gradual declining trend over the years [26,27]. Meanwhile, *Microcystis* can rapidly accumulate large amounts of phosphorus as polyphosphate (Poly-P) through luxury uptake under phosphorus-replete conditions [28]. This mechanism can lead to the rapid depletion of dissolved inorganic phosphorus (DIP) to very low levels following algal blooms, even in originally phosphorus-rich eutrophic waters, resulting in a localized phosphorus-limited environment [28]. Nevertheless, some *Microcystis* species, such as *M. aeruginosa* and *Microcystis densa*, can not only survive under such P-deficient conditions for extended periods but also maintain exponential growth [29,30]. The aforementioned remediation measures centered on nutrient control do not necessarily mitigate the presence of emerging contaminants in aquatic environments; therefore, the impact of such contaminants (e.g., pharmaceuticals) cannot be overlooked. Consequently, exposure to antibiotics and limitation by P may occur simultaneously. However, little research has been conducted on the adaptive strategies of cyanobacteria under antibiotics/P-deficiency co-stress, especially at the gene regulatory level.

The aim of this study was to determine the response of colonial cyanobacteria to antibiotics/P-deficiency co-stress. An antibiotic exposure experiment was conducted by using colonial *M. aeruginosa*: (1) to examine the toxicity effects of ETM on colonial cyanobacterium *M. aeruginosa* under nutrient-replete and phosphorus-limited conditions by measuring cell proliferation, Fv/Fm, chlorophyll *a* (Chl. *a*), activities of antioxidant enzymes, EPS and algal cytoplasmic membrane potential; (2) to unravel the toxicological mechanism of ETM on algal growth under different nutrient conditions via metatranscriptomic and metabolomic approaches.

## 2. Materials and Methods

### 2.1. Algal Culture and Growth Conditions

In this study, we used a strain of colonial morphotype of *M*. *aeruginosa*, which was supplied by the Freshwater Algae Culture Collection of the Institute of Hydrobiology, Chinese Academy of Sciences (No. FACHB-2442). Before the exposure experiment, the strain was precultured for 2 weeks in sterile BG11 medium at 25 °C under a 12 h:12 h day-and-night cycle with a white light intensity of 30 μmol·photons/m^2^·s to maintain its colonial morphology.

### 2.2. Exposure Experiment

ETM (CAS: 114-07-8) was purchased from ANPEL Laboratory Technologies (Shanghai) Inc. (Shanghai, China), with a purity of ≥98.4%. ETM was dissolved in dimethyl sulfoxide (DMSO) and stored in the refrigerator at −20 °C as stock solutions and then diluted with ultrapure water to obtain the intermediate solutions. Cyanobacterial cells in logarithmic growth phase were inoculated at 2 × 10^5^ cells/mL into 150 mL sterile medium under normal-BG11 (BG11) and phosphorus-limited-BG11 (LP, 0.02 mg/L P) conditions. To deplete intracellular P reserves, the algal colonies of the LP treatments were washed three times with P-free sterile BG11 medium and then cultured in the P-free medium for ten days before the exposure experiment [31]. Every flask was shaken three times daily. The cells were exposed to ETM for 12 days. The concentrations of ETM were determined as 0 (solvent control), 0.01, 0.1, 1, 10, 20 and 100 μg/L through preliminary tests. Final concentration of the organic solvent (DMSO) in the culture medium of each treatment was controlled within 0.05‰ (*v*/*v*). During the test, ETM would undergo photolysis and hydrolysis [1]. Throughout the experiment, the ETM concentration was consistently controlled within the predetermined range to maintain pseudo-persistent contamination conditions (Figure S1). In the experiment, the labels use B and L to represent the two nutrient levels, and the numbers following them indicate the concentration of ETM. Each treatment had three biological replicates.

### 2.3. Cellular Growth Counting

One milliliter of the algal culture was sampled from each flask to calculate the cell density using the Flow Camera and Microscope (Flowcam 8400; Yokogawa Fluid Imaging Technologies, Scarborough, ME, USA). Before calculation, the colonial morphotypes were disintegrated using 0.05 M sodium hydroxide at 85 °C for 8 min [32].

### 2.4. Photosystem and Antioxidant Analyses

The fluorescence parameter Fv/Fm was a target parameter that corresponded to the maximum photochemical quantum yield of the phytoplankton Photosystem (PSII). Two milliliters of the algal culture were sampled from each replicate of each treatment to analyze the Fv/Fm and cellular Chl. *a* by Pulse-Amplitude-Modulation Chlorophyll Fluorometer (PHYTO-PAM-II Modular; Heinz Walz, Effeltrich, Germany) every two days. The cultures were dark-adapted for 15 min before measuring.

To investigate the influence of ETM on the antioxidant system of colonial *M. aeruginosa*, the activities of two antioxidant enzymes, Superoxide Dismutase (SOD) and Catalase (CAT), were measured using corresponding assay kits (Nanjing Jiancheng Bioengineering Institute, Nanjing, China) following the manufacturer’s standard protocols, respectively.

### 2.5. Extracellular Polymeric Substance Analysis

It is known that the EPS contains polysaccharides, proteins, and lipids. In this study, the quantities of extracellular polysaccharides and proteins were measured. Lipids were not quantified because they usually account for only a minor proportion of algal EPS. In addition, Deng et al. indicated that their direct contribution to the aggregation of algal cells is limited relative to that of polysaccharides and proteins [33]. The EPS extraction was performed according to Xu et al. [34]. The polysaccharide content was determined using the phenol-sulfuric acid method, and protein contents were determined with bovine serum albumin as the standard according to Deng et al. [35]. The total EPS content was calculated as the sum of the polysaccharide and protein concentrations.

### 2.6. Cyanobacterial Plasma Membrane Potential and Colony Size Analyses

The plasma membrane potential is a typical indicator that can be used to characterize the cellular response under toxic exposure. In this study, the plasma membrane potential of *M. aeruginosa* was estimated by the 3,3′-Dihexyloxacarbocyanine iodide potentiometric probe (DiOC_6_(3), CAS: 53213-82-4; Sigma-Aldrich, Co., Steinheim am Albuch, Germany). A total of 25 μM DiOC_6_(3) was added to one milliliter of the *M. aeruginosa* culture at room temperature for 8 min in darkness before analysis [36].

To examine the influence of ETM on the morphology of cyanobacteria, one milliliter of the algal culture was sampled from each replicate of each treatment to analyze colony size by a Zeiss Axioskop microscope (Carl Zeiss, Oberkochen, Germany) at 40× or 100× magnification.

### 2.7. Metabolite Extraction and Metabolomic Analysis

The six treatments of samples (B0, B0.01, B1, B10, L0, and L10) were transferred to injection vials after extraction and analyzed using an ultra-performance liquid chromatograph (ACQUITY UPLC I-Class plus; Waters, Milford, MA, USA) linked to a high-resolution mass spectrometer (QE HF; Thermo Fisher, Waltham, MA, USA) with a chromatographic column (ACQUITY UPLC HSS T3; 100 mm × 2.1 mm, 1.8 μm, Waters, Milford, MA, USA). The metabolomic analysis was performed by Shanghai OE Biotech Co., Ltd. (Shanghai, China). The detailed extraction procedures and data analysis processes are provided in Text S1.

### 2.8. Total RNA Extraction and Metatranscriptomic Analysis

Metatranscriptomic analysis of six groups (B0, B0.01, B1, B10, L0, and L10) was performed by Guangzhou Magigene Biotechnology Co., Ltd. (Guangzhou, China). Briefly, total RNA was extracted and used for library preparation, followed by paired-end sequencing on an Illumina HiSeq platform. Subsequent steps included quality control of raw data, gene abundance quantification, and functional annotation based on the KEGG database for downstream analysis. Detailed experimental and analytical procedures are provided in Text S2.

### 2.9. Statistical Analysis

Data are expressed as means ± standard deviation (SD) of three biological replicates. All graphs were generated using OriginPro 2021 (OriginLab, Northampton, MA, USA). Statistical analyses were performed using IBM SPSS Statistics 27 (IBM Corp., Armonk, NY, USA). For variables measured repeatedly over time (cell density, Fv/Fm, and Chl. *a*), repeated-measures ANOVA was conducted with treatment as the between-subjects factor and time as the within-subjects factor. For endpoint measurements (antioxidant enzyme activities, EPS, membrane potential, and colony size), one-way ANOVA was employed for multi-group comparisons. Prior to ANOVA, normality was checked using the Shapiro–Wilk test and homogeneity of variances was assessed using Levene’s test. Data that violated the assumption of homogeneity of variances were analyzed using Welch’s correction. For post hoc comparisons, Tukey’s HSD test was applied for one-way ANOVA, and Bonferroni correction was applied for repeated-measures ANOVA. For metabolomic data, differential metabolites were identified based on the criteria of *p* < 0.05 and |log_2_FC| ≥ log_2_(1.2), as detailed in Text S1. For metatranscriptomic data, differentially expressed genes (DEGs) were identified using |log_2_FC| ≥ 1 and FDR < 0.05 as the cutoff criteria. KEGG pathway enrichment analyses for both metabolomic and metatranscriptomic data were performed using Fisher’s exact test (equivalent to the hypergeometric test), with FDR < 0.05 considered statistically significant. In all cases, a *p*-value < 0.05 was considered statistically significant.

## 3. Results

### 3.1. Effects of ETM on Cell Proliferation

**Figure 1:**
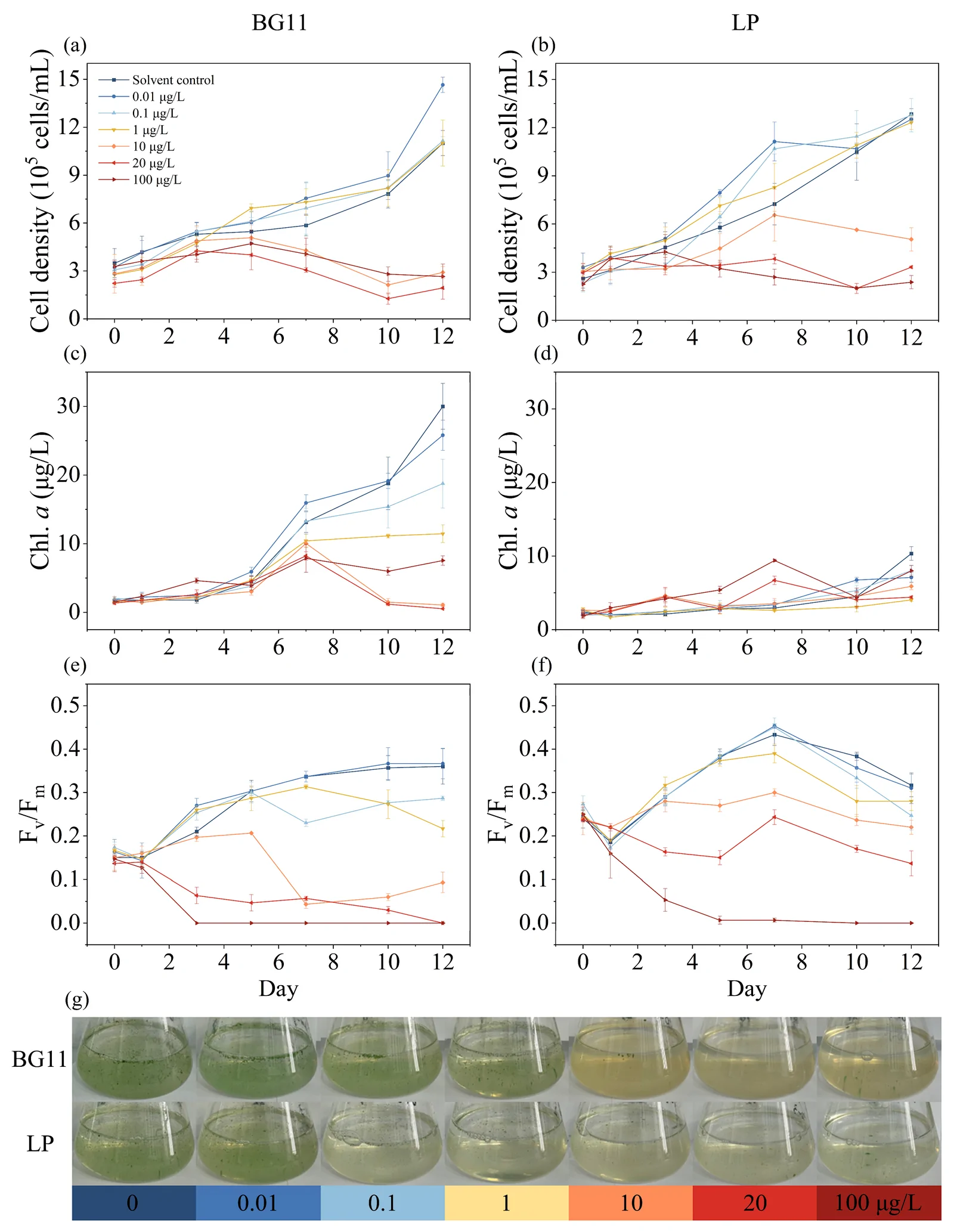
Effects of erythromycin (ETM) on cell density under normal-BG11 (BG11) (**a**) and phosphorus-limited-BG11 (LP) (**b**) culture conditions. Effects of ETM on Chl. a under BG11 (**c**) and LP (**d**) culture conditions. Effects of ETM on Fv/Fm under BG11 (**e**) and LP (**f**) culture conditions. Images of each culture group of colonial *Microcystis aeruginosa* after 12 days of growth (**g**).

The physiological response of colonial *M. aeruginosa* to ETM/P co-stress was tested under different conditions of ETM and P. Figure 1 reveals that the growth of colonial *M. aeruginosa* was affected by ETM under both BG11 and LP conditions. Low concentrations of ETM (0.01–1 μg/L) in BG11 conditions had a hormesis effect on the growth of colonial *M. aeruginosa*. Their growth was significantly inhibited at the high ETM exposure concentrations (10–100 μg/L) under both BG11 and LP conditions (*p* < 0.01).

### 3.2. Effects of ETM on the Photosystem

The photosynthetic performance was significantly influenced by different concentrations of ETM. Except for the 0.01 μg/L treatment, all other treatments exhibited an inhibitory effect after exposure to ETM for 12 days (Figure 1e,f). High concentrations, especially the 100 μg/L treatments, significantly decreased the Fv/Fm (*p* < 0.01). After culture for 3 days, Fv/Fm had decreased to 0 under BG11 conditions. Compared with BG11 treatments, LP treatments showed a slower decrease in Fv/Fm. Compared to the initial value (day 0), the Fv/Fm in the B10 treatment decreased by 37.78% after 12 d exposure, whereas it decreased by only 8.33% in the L10 treatment. The significant reductions in Fv/Fm observed during the experiment showed no recovery after prolonged ETM exposure in the affected subgroups, indicating irreversible damage to PSII in these cases. Such damage ultimately led to the growth inhibition of *M. aeruginosa*. Chlorophyll *a* showed an overall increasing trend, with the BG11 treatments increasing more rapidly. However, each treatment displayed a noticeable inhibitory response. Overall, these results consistently showed that ETM had a negative effect on the photosystem of colonial *M. aeruginosa*.

### 3.3. Effects of ETM on the Antioxidant System

**Figure 2:**
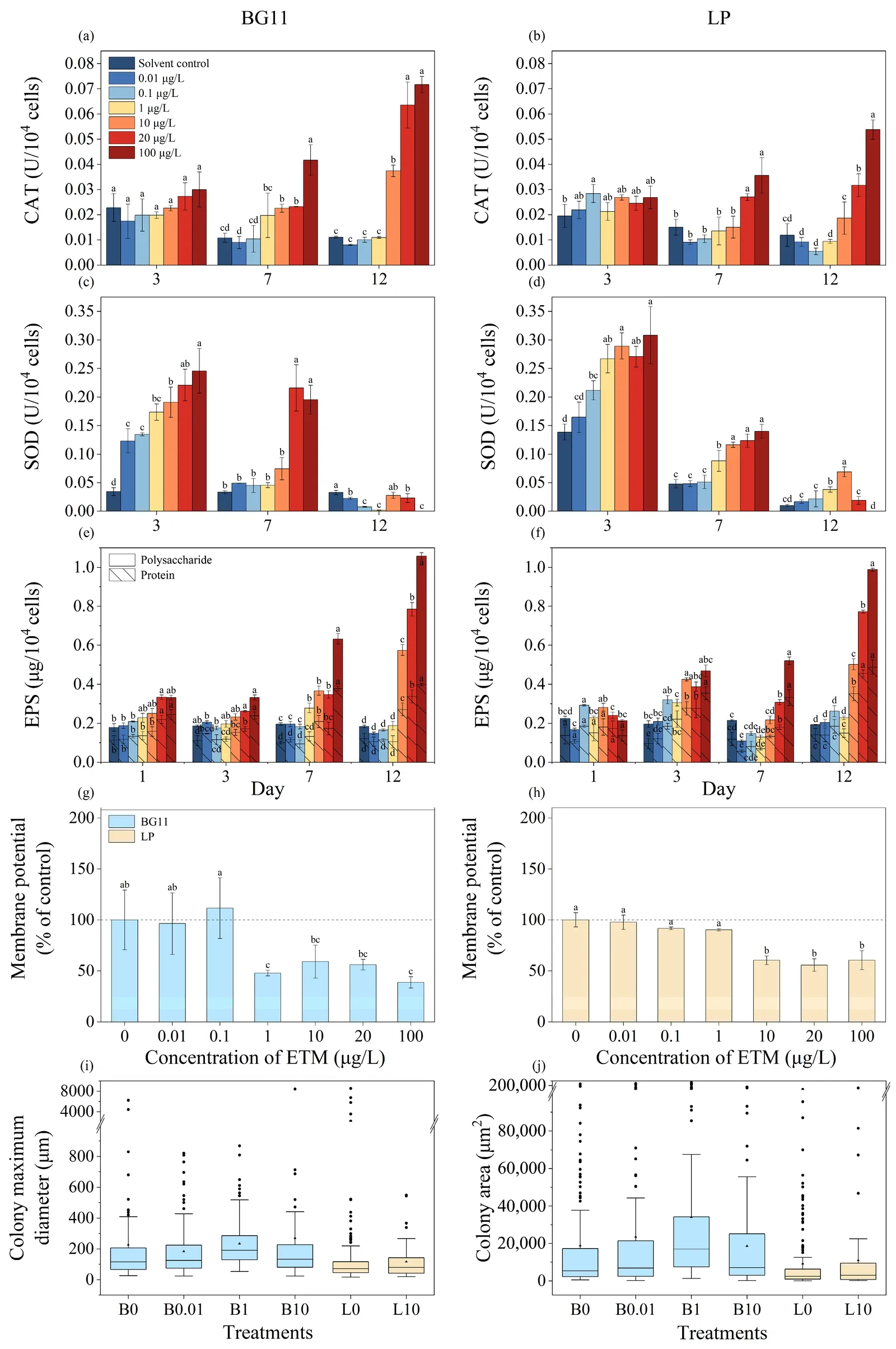
Effects of erythromycin (ETM) on CAT under normal-BG11 (BG11) (**a**) and phosphorus-limited-BG11 (LP) (**b**) culture conditions. Effects of ETM on SOD under BG11 (**c**) and LP (**d**) culture conditions. Effects of ETM on EPS under BG11 (**e**) and LP (**f**) culture conditions. Plasma membrane potentials of colonial *Microcystis aeruginosa* exposed to different concentrations of ETM after 12 d under BG11 (**g**) and LP (**h**) culture conditions. Results are given as percentage DiOC6(3)-derived MFI of solvent control treatment. Effects of ETM on colony maximum diameter (**i**) and colony area (**j**) under BG11 and LP culture conditions. Different lowercase letters (a, b, c, …) indicate significant differences among treatments at *p* < 0.05, while the same letters indicate no significant difference.

CAT and SOD are common indicators of antioxidant response in algae and other organisms after exposure to certain contaminants that trigger the formation of oxygen radicals [37]. Overall, the CAT activities tended to increase as the concentration of ETM increased. After 7 days or 12 days of exposure to ETM, the CAT activities significantly increased at ETM concentrations of 10, 20 and 100 μg/L (*p* < 0.01) (Figure 2). Compared with the solvent control treatment, no significant differences were observed at 0.01–1 μg/L after 12 days of exposure. Relative to the BG11 culture condition, the high-concentration ETM treatments under LP culture condition exhibited lower CAT activities over the same exposure period. Similar to CAT activities, SOD activities exhibited an upward trend with an increase in ETM concentration. However, significant differences in SOD activities among different treatments emerged after 3 days of exposure, which was earlier than CAT activities. The SOD activities at ETM concentrations of 0.01 to 10 μg/L were significantly increased relative to those of the solvent control treatment at 3 days (*p* < 0.01) but displayed no significant difference at 7 days. The SOD activities significantly increased at ETM concentrations of 20 and 100 μg/L throughout the first 7 days of exposure (*p* < 0.01).

### 3.4. Effects of ETM on the EPS

The content of EPS generally increased with the increase in ETM concentration. It significantly increased at ETM concentrations of 10, 20 and 100 μg/L compared with the solvent control treatment (*p* < 0.01), achieving 2.13, 3.27 and 4.76 times under BG11 culture conditions and 1.61, 3.00 and 4.11 times under LP culture conditions at 12 days, respectively (Figure 2). Compared to the BG11 treatments, the LP treatments exhibited reductions in EPS increases of 24.41%, 8.26%, and 13.66% at the corresponding concentrations, respectively. The result indicates that phosphorus deficiency reduces EPS production. Within the composition of EPS, polysaccharides exhibited a higher rate of increase.

### 3.5. Effects of ETM on the Cyanobacterial Plasma Membrane Potential

Distinct alterations of plasma membrane potential were detected after 12 days (Figure 2). Obvious differences were observed between concentrations of 0–0.1 μg/L and 1–100 μg/L under BG11 culture conditions (*p* < 0.01), and between 0 and 1 μg/L and 10 and 100 μg/L under LP culture conditions (*p* < 0.01).

### 3.6. Effects of ETM on the Colony Size

The size of *M. aeruginosa* colonies (maximum diameter and area) generally increased and then decreased with the increase in ETM concentration under BG11 culture conditions, with the maximum size at an ETM concentration of 1 µg/L (Figure 2). In addition, the sizes of the colonies at the phosphorus-limited treatments were significantly smaller than those of the BG11 treatments (*p* < 0.05), indicating that the phosphorus-limited environment has reduced the size of algal aggregates.

### 3.7. Metabolomic Analysis

**Figure 3:**
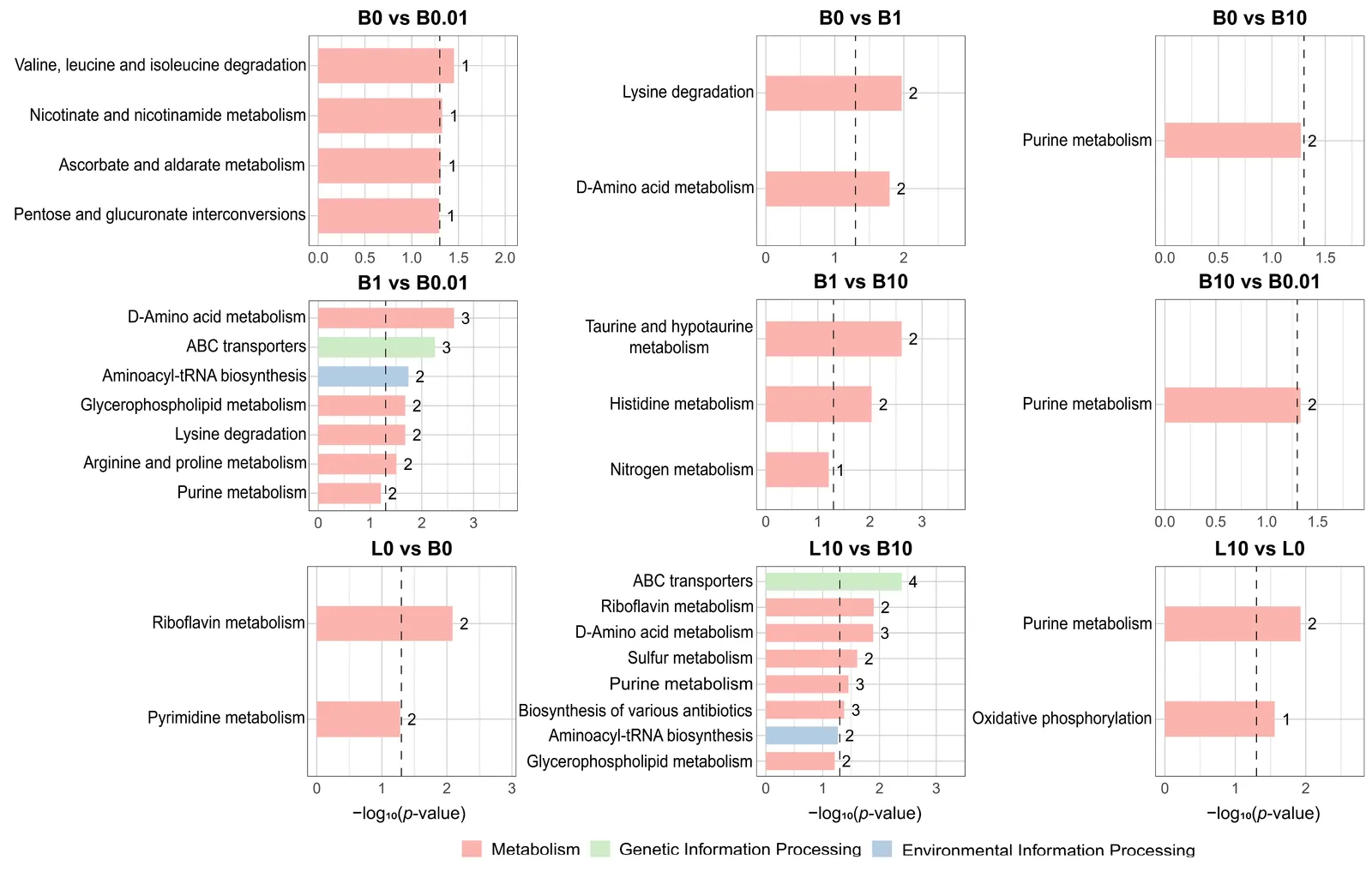
KEGG enrichment analysis of differential metabolites of *Microcystis aeruginosa* under different treatments. The *x*-axis represents the enrichment significance expressed as −log_10_(*p*-value), and the *y*-axis lists the names of KEGG pathways at Level 3. The numbers on the bars indicate the count of differential metabolites annotated to each pathway, and the different bar colors represent the corresponding KEGG Level 1 categories. The dashed vertical line indicates the significance threshold at *p* = 0.05; bars to the left of this line correspond to pathways with *p* > 0.05, while bars to the right indicate pathways with *p* < 0.05.

A total of 1909 metabolites were identified among the six cyanobacterial treatments. These metabolites were classified into nine categories at the class level by Chemical Taxonomy of HMDB, primarily including Benzenoids and Substituted Derivatives, Carboxylic Acids and Derivatives, Fatty Acyls, Glycerolipids, Glycerophospholipids, Organonitrogen Compounds, Organooxygen Compounds, Prenol Lipids, and Steroids and Steroid Derivatives (Figure S2a). Most metabolites (1816 in total) were detected across all treatments, indicating that 95% of these metabolites were shared (Figure S2b). As shown in Figure S3a, PCA results revealed clear separation between the BG11 and LP treatments, and between the 10 μg/L group and other concentration groups using BG11 medium. Volcano plots were generated for comparisons between different treatment groups (Figure S4), with the numbers of the differential metabolites (DMs) identified summarized in Table S2. Notably, among the 243 DMs identified between B0 and B1 treatments, 187 DMs were upregulated and 56 DMs downregulated in B1. Between B0 and B10 treatments, 82 DMs were upregulated and 236 DMs downregulated in B10. Between L10 and B10 treatments, 317 DMs were upregulated and 263 DMs downregulated in L10. Furthermore, between the L10 and B10 treatments, 580 DMs were identified, with 317 upregulated and 263 downregulated in the L10 treatment. Further visualization through heatmap analysis ranked by the top 50 relative abundance across groups revealed distinct metabolic patterns (Figure S5). As shown in Figure 3, we identified in the KEGG database that the screened DMs among all the above treatments are primarily involved in metabolism, environmental information processing, and genetic information processing. Among them, the DMs between the B0 and B1 treatments were mainly involved in processes such as lysine biosynthesis, D-amino acid metabolism, and biotin metabolism; the DMs between the B0 and B10 treatments were mainly involved in processes such as purine metabolism, valine, leucine, and isoleucine biosynthesis, and pyruvate metabolism; and the DMs between the L10 and B10 treatments were mainly involved in processes such as ABC transporters, riboflavin metabolism, and D-amino acid metabolism.

### 3.8. Metatranscriptomic Analysis

A total of 636,467 genes were identified across the six cyanobacteria treatments. These genes were ranked according to their relative abundance, and the top 500 genes were selected for further analysis. The heatmap of the top 500 genes across six treatment groups is depicted in Figure S6. The BG11 and LP treatments exhibited completely opposite gene expression trends, indicating that phosphorus content could significantly alter the gene expression of *M. aeruginosa*. Moreover, under the same nutrient level, the gene expression in the ETM concentration of 10 μg/L showed some degree of variation compared to control treatments, suggesting that exposure to high concentrations of ETM can modify the gene expression of *M. aeruginosa*. The differences among treatment groups were evaluated using PCA. Consistent with the metabolomic results, a clear separation was observed between the two nutrient-level treatments (Figure S7).

DEGs were screened from 636,467 genes in *M. aeruginosa* cells exposed to different concentrations of ETM, visualized in volcano plots (Figure S8). DEGs in B10 vs. B0 increased 270-fold relative to B1 vs. B0, with 89.7% (4608 genes) downregulated. This suggests that high ETM concentrations may overcome cellular defense mechanisms and induce widespread transcriptional suppression. Additionally, phosphorus limitation induced significant differential expression of 24,661 genes (L10 vs. B10), of which 63.7% were upregulated. It demonstrates that phosphorus limitation conditions improve the stress response to high concentrations of ETM by activating adaptive transcriptional programs.

**Figure 4:**
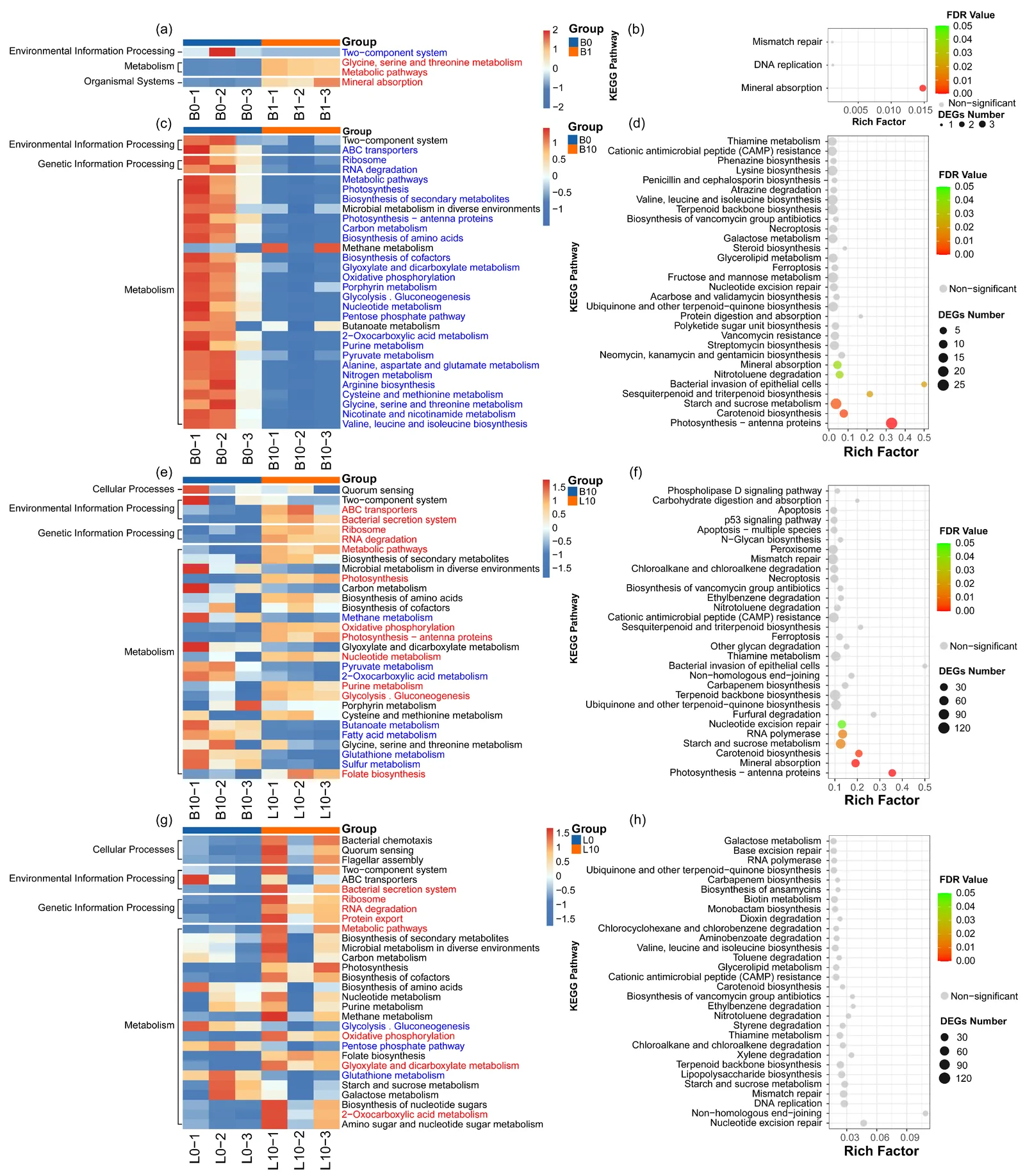
KEGG enrichment analysis of differentially expressed genes of *Microcystis aeruginosa* under different treatments. Heatmap of Level 3 pathway abundance in the B0 vs. B1 (**a**), B0 vs. B10 (**c**), B10 vs. L10 (**e**), and L0 vs. L10 (**g**) comparisons. The left and right y-axes indicate the KEGG Level 1 classification and Level 3 pathway names, respectively. Blue annotated pathways denote significantly lower abundance (*p* < 0.05), red indicates significantly up-expressed pathways (*p* < 0.05), and black marks non-significant differences (*p* > 0.05). Bubble plot of differential KEGG enrichment analysis (**b**,**d**,**f**,**h**). *X*-axis: Enrichment factor (differentially expressed genes/total annotated genes). *Y*-axis: KEGG pathways. Bubble size corresponds to the relative quantity of enriched differentially expressed genes; color gradient reflects FDR (green-red: FDR < 0.05 by Fisher’s exact test; grey: non-significant).

Further functional annotation and clustering of genes were performed. The KEGG relative abundance results for significantly differentially expressed genes at Level 3 are shown in Figure 4a,c,e,g. Compared to the control treatment B0, B1 showed higher abundances of pathways associated with organismal systems, environmental information processing and metabolism. Among these, Mineral absorption was significantly enriched (FDR < 0.05) and upregulated (*p* < 0.05). In B10 vs. B0, pathways include core metabolism and energy-related pathways such as Photosynthesis, Photosynthesis-antenna proteins, Carbon metabolism, Oxidative phosphorylation, and Nitrogen metabolism, as well as genetic information processing such as Ribosome and RNA degradation, which showed significant downregulation (*p* < 0.05). Additionally, environmental information processing pathways related to environmental adaptation and stress resistance, including Two-component system and ABC transporters, also exhibit relatively lower abundance. Among them, Photosynthesis-antenna proteins were significantly enriched (FDR < 0.05), with 13 related genes showing downregulation (Figure S9). In contrast to the widespread downregulation induced by high-concentration ETM (B10 vs. B0), the L10 vs. B10 comparison showed significant upregulation (*p* < 0.05) of multiple pathways in L10, including Metabolic pathways, Photosynthesis, Oxidative phosphorylation, Photosynthesis-antenna proteins, ABC transporters, Bacterial secretion system, Ribosome, RNA degradation, and Folate biosynthesis. Conversely, pathways such as Methane metabolism, Pyruvate metabolism, 2-Oxocarboxylic acid metabolism, Butanoate metabolism, Fatty acid metabolism and Sulfur metabolism showed significant downregulation (*p* < 0.05). Photosynthesis-antenna proteins remained a significantly enriched pathway (FDR < 0.05), with 14 related genes showing upregulation (Figure S9). Moreover, it is noteworthy that under phosphorus-limited conditions, Phosphonate and phosphinate metabolism showed significant upregulation (*p* < 0.05). Specifically, the enzyme alpha-D-ribose 1-methylphosphonate 5-phosphate C-P lyase (EC: 4.7.1.1) within this pathway was upregulated (Figure S10). Meanwhile, the relative abundance of the Phosphonate and phosphinate metabolism pathway increased by 7663.47% in L0 versus B0, and by 76,868.66% in L10 versus B10, respectively. Notably, our metatranscriptomic data revealed that programmed cell death pathways (e.g., ko04215, Apoptosis-multiple species) [38] were not significantly enriched in low- and high-concentration ETM treatments.

## 4. Discussion

### 4.1. ETM Exerts Hormesis Effects on M. aeruginosa by Stimulating Growth at Low Levels and Damage Effects at High Levels

This study revealed that ETM exerts concentration-dependent effects on *M. aeruginosa*, displaying a hormetic response consistent with previous findings on unicellular cyanobacteria [32]. Wu et al. found that the Fv/Fm of unicellular *M. aeruginosa* only decreased significantly when ETM exceeded 10 μg/L [39]. In contrast, our study showed a significant decrease in colonial *M. aeruginosa* at 0.1 μg/L, indicating that the photosynthetic system of colonial algae is more sensitive to ETM than that of unicellular algae. Correspondingly, transcriptomic analysis indicated that low ETM exposure upregulated genes associated with mineral absorption. This suggests that ETM may enhance the uptake of essential metal ions such as iron and magnesium, thereby promoting PSII activity and cell growth [40,41]. In this study, elevated SOD and CAT activities after 3 and 7 days of exposure indicated that mild oxidative stress triggered the activation of the antioxidant defense system [42,43]. The upregulation of metabolites such as oxidized phospholipids (e.g., PA(8:0/20:4+=O)) and gingerol derivatives (e.g., [8]-Shogaol) further indicated an increase in ROS levels and a metabolic response to this regulation [44]. Meanwhile, the significant increase in antioxidant potential [40,41] piperitol [45], and L-lysine, known to suppress ROS production [46], indicate that *M. aeruginosa* cells activated their metabolic networks to mount an active defense response. Morphologically, a bell-shaped (inverted U) relationship between colony size and ETM concentration was observed, providing new insights into the potential effects of antibiotics on harmful algal blooms. Low concentrations of antibiotics may stimulate *M. aeruginosa* to secrete EPS, inducing biofilm formation and potentially facilitating the development of larger colonies through enhanced cell aggregation [47]. EPS can react with hydroxyl radicals (OH·), delaying contact between the target antibiotic and cells, thereby protecting the cell membrane [48]. Therefore, its production is also a manifestation of the self-protection mechanism of cyanobacteria [22,49]. These findings suggest that at low ETM concentrations (0.01–1 μg/L), ETM enhances the uptake of trace elements by *M. aeruginosa*, strengthens its carbon metabolism, and increases its synthesis of proteins, polysaccharides, and chlorophyll precursors. These processes support orderly cell-cycle progression, including DNA replication and division-related protein synthesis, ultimately promoting cell proliferation [50].

High ETM concentrations inhibited growth through photosynthesis suppression, oxidative damage, and membrane dysfunction. At high ETM treatments, Fv/Fm and Chl. *a* declined markedly, suggesting rapid inhibition of PSII and chlorophyll synthesis. Correspondingly, transcriptomic analysis revealed significant downregulation of genes related to photosynthesis and photosynthesis-antenna proteins, which may be because high-concentration ETM may induce excessive accumulation of ROS, thereby blocking electron transfer at the PSII reaction center, inhibiting chlorophyll synthesis, and impairing light-harvesting efficiency [6]. Concurrently, downregulation of photosynthesis-related genes and suppression of oxidative phosphorylation led to insufficient energy supply, further exacerbating cell cycle arrest, ultimately manifesting as growth inhibition and reduced PSII activity [51]. At high ETM treatments, SOD and CAT activity could not neutralize ROS levels, resulting in disrupted intracellular homeostasis and a significant decline in SOD and CAT activity [42,52]. Sustained elevation of oxidized phospholipids (PA(8:0/20:4+=O)) in high-concentration treatments indicates that excessive ROS attacks membrane structures, leading to elevated lipid peroxidation and further compromising cell membrane permeability [53]. High-concentration ETM induced algal cell depolarization, resulting from inhibition of the proton pump (H^+^-ATPase) activity and subsequent K^+^ efflux, exacerbating the electrochemical imbalance, ultimately culminating in lysis [54,55]. We posited that ETM-induced plasma membrane damage likely stems from disruption of plasma membrane-associated proteins [46]. In addition, significant downregulation of nicotinamide suggested disruption of NAD^+^ synthesis and inhibited energy metabolism, which would restrict cell growth [56,57]. Transcriptomic analysis revealed that ribosomal pathways, purine metabolism, and pyrimidine metabolism were all markedly downregulated. Insufficient nucleotide supply directly limited the efficiency of DNA replication, while inhibition of protein translation affected the synthesis of functional and structural proteins required for each phase of the cell cycle, thereby blocking the cell cycle at multiple points [58].

### 4.2. Phosphorus Deficiency Enhances the Tolerance of M. aeruginosa to ETM

**Figure 5:**
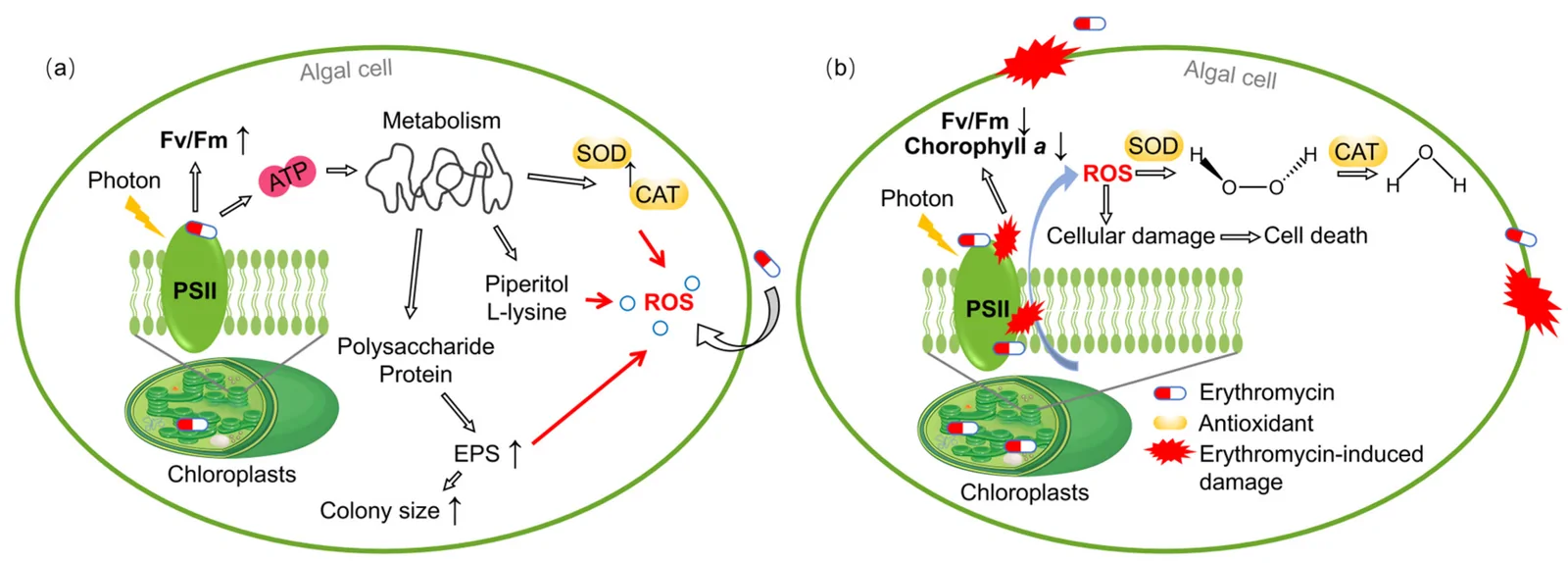

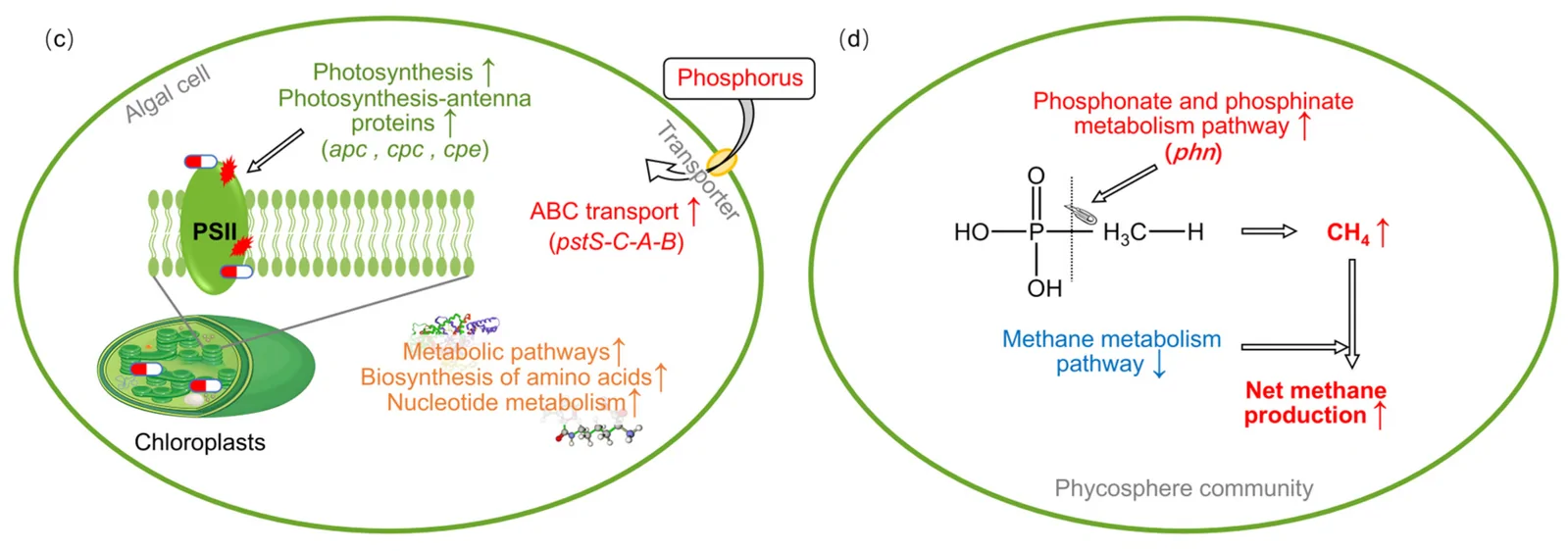
Schematic representation of the toxicological effects of low-concentration erythromycin (ETM) on *Microcystis aeruginosa* (**a**). Schematic representation of the toxicological effects of high-concentration ETM on *M. aeruginosa* (**b**). Schematic representation of the resistance process of *M. aeruginosa* to ETM under phosphorus limitation conditions (**c**). Schematic representation of the mechanism for enhanced net methane formation potential under phosphorus limitation (**d**).

This study observed that the low-P environment increased both the ETM concentration threshold and the exposure time required to significantly reduce algal cell density. (Figure 5) Compared with B10, the enhanced tolerance of the photosynthetic system in L10 may be associated with impacts on the algal antioxidant system. During the initial phase of P deficiency (within 3 days), SOD activity showed a slight increase relative to the BG11 treatments, which may reduce the toxicity of ROS to Fv/Fm caused by ETM. The result aligns with findings by Qiu et al. [23], indicating that P limitation can enhance cyanobacterial tolerance to superoxide radicals (O_2_^−^), thereby potentially reducing ROS-induced damage to PSII. Compared with B10, L10 showed altered levels of long-chain acylglycines (upregulation of n-undecylglycine) and fatty acid amides (upregulation of erucamide, downregulation of dodecanamide and myristamide). These changes reflect a shift from short-chain to long-chain derivatives, adapting to metabolic constraints imposed by P limitation. Under P-limiting conditions, *M. aeruginosa* demonstrates multidimensional adaptive strategies through metabolic reprogramming, for example, the upregulation of long-chain unsaturated fatty acids [59]. Moreover, identified erucamide is an antibacterial phytoalexin that inhibits competitor growth (e.g., other algae or bacteria) [60], which may represent an ecological niche defense strategy and enhance *M. aeruginosa*’s competitiveness in resource-limited conditions. Notably, the accumulation of potential bioactive secondary metabolites propyl 2,4-decadienoate and pipericine in L10 reflected a metabolic reallocation strategy of *M. aeruginosa* under ATP synthesis limitation caused by P deficiency [61]. However, this defense strategy carries a fitness cost. In conjunction with the transcriptomic evidence of downregulated ribosomal pathways and purine metabolism, as well as reduced growth rates and PSII efficiency, we propose that under multiple stressors, algal cells prioritize defensive metabolism at the expense of their own cell division and proliferation.

The P-limited environment enhanced cyanobacterial tolerance to ETM via precise energy production and reallocation strategies. Compared with B10, the comprehensive upregulation of the photosynthesis and photosynthesis-antenna protein pathways (with significant upregulation of genes related to phycobiliprotein synthesis such as *apcA-F*, *cpcA-G*, and *cpeS*) indicates that algae are compensating for potential declines in metabolic efficiency by enhancing light capture capability [62]. Meanwhile, significant upregulation of pathways including metabolic pathways, biosynthesis of amino acids, nucleotide metabolism, purine metabolism, and ribosome in L10 indicates reinforced biosynthetic capacity for fundamental cellular components (amino acids, nucleotides, proteins) to sustain viability under P stress. The upregulation of the high-affinity inorganic phosphate (Pi) transporter system (*pstS-C-A-B*) drove activation of the ABC transporter pathway (Figure S11). Enhanced expression of *pstS* (encoding the periplasmic phosphate-binding protein), *pstA* (encoding the transmembrane channel protein), and *pstB* (encoding the ATPase) directly responds to low-P conditions to improve Pi uptake efficiency, signifying enhanced transmembrane transport capacity to combat P scarcity [63,64]. In contrast, cells downregulated pyruvate metabolism, glyoxylate cycle, and fatty acid metabolism to restrict non-essential metabolic processes. They also actively attenuated antioxidant defense systems, exemplified by downregulation of glutathione metabolism and sulfur metabolism, thereby redirecting resources toward core functions. This transcriptional pattern aligns with observed reduced CAT activity under P limitation. The overall upregulation and downregulation of pathways reveal that algae, under combined ETM and phosphorus stress, adapt by sacrificing some flexibility in carbon metabolism while enhancing basic synthesis, energy supply, and key nutrient uptake (such as phosphate transport) to maintain cellular homeostasis.

### 4.3. Phosphorus Deficiency May Enhance the Net Methane Formation Potential

In this study, KEGG enrichment analysis of the metatranscriptome revealed that compared with B0, the relative abundance of the phosphonate and phosphinate metabolism pathway in L0 increased by 77.63-fold. This finding corroborates previous studies and indicates that under phosphorus-limited conditions, cyanobacterial phycosphere communities can utilize environmental methylphosphonates as a phosphorus source via sustained expression of the *phn* gene cluster, indirectly producing methane [65,66]. Concurrently, the methane metabolism pathway in L0 vs. B0 was downregulated to 0.34-fold, leading to increased methane accumulation within the system. We further compared the B10 and L10 treatments under combined stress of phosphorus limitation and ETM. Similar to the comparisons between L0 and B0, the L10 treatments showed a significant upregulation (*p* < 0.05) of phosphonate and phosphinate metabolic pathways compared with B10, with relative abundance increased by 770-fold. Methane metabolism was downregulated to 0.29-fold. In summary, from a metatranscriptomic perspective, we observed that phosphorus-limited conditions may increase the net methane production in the culture system.

In our experiment, after some cell death and rupture under ETM stress, the release of cellular contents increased the organic phosphorus content in the environment, which is the main source of C-P cleavage in *phnJ* and other genes under BG11 conditions [67]. Notably, in the management of large freshwater eutrophic lakes such as Tai Lake and Chao Lake, restricting phosphorus inputs has not yielded effective control of cyanobacterial blooms. Instead, the frequency and intensity of blooms have increased over recent decades [68]. During bloom periods, abundant nitrogen enhances cyanobacterial phosphorus uptake capacity, leading to a rapid depletion of bioavailable Pi [69], thereby creating conditions conducive to *phnJ* expression. Furthermore, widespread glyphosate pollution in freshwater systems provides a substantial source of methylphosphonates, which could potentially amplify methane production by 4- to 10-fold [66]. Therefore, controlling cyanobacterial blooms cannot rely solely on phosphorus reduction; nitrogen management and organic phosphate pollutant control should also be considered.

## 5. Conclusions

Colonial *M. aeruginosa* exhibits a biphasic response to ETM. Low concentrations (≤1 μg/L) promote cell proliferation and colony enlargement of colonial *M. aeruginosa*. In contrast, high concentrations (≥10 μg/L) inhibit photosynthesis, induce oxidative stress, stimulate EPS secretion, and disrupt membrane potential. Low ETM concentrations promote *M. aeruginosa* growth via improved nutrient assimilation and biosynthetic activity, while high concentrations inhibit photosynthesis and ribosome function, causing oxidative damage, membrane disruption, and eventual lysis. Phosphorus limitation may enhance tolerance to ETM through metabolic reallocation, reinforced phosphate transport (upregulated *pstS-C-A-B*), and optimized light harvesting (upregulated *apcA-F*). Our metatranscriptomic data suggest that P-limited conditions may increase the net methane production potential by increasing the relative abundance of the phosphonate and phosphinate metabolism pathway while reducing that of methane metabolism. These findings underscore that assessing the ecological risks of antibiotics and developing control strategies for lake eutrophication must comprehensively consider the low-dose stimulatory effects and phosphorus limitation-mediated cross-tolerance.

## Data Availability

The raw reads were deposited in the National Omics Data Encyclopedia (NODE) (https://www.biosino.org/node/ accessed on 5 November 2025) under Sample ID OEP00006676.

## Supplementary Materials

The following Supporting Information can be downloaded at: https://www.mdpi.com/article/10.3390/toxics14080660/s1, Text S1. Detailed extraction procedures and data analysis processes for metabolomic analysis; Text S2. Detailed experimental and analytical procedures for metatranscriptomic analysis; Table S1. PERMANOVA analysis of metabolites at different treatments; Table S2. The numbers of differential metabolites; Figure S1. The variation of the determined concentrations of erythromycin after 12 days of culture at nominal concentration of 1, 10, 20 and 100 μg/L, with a regular replenishment of erythromycin. Mean ± standard deviation of three replicates is shown for each value; Figure S2. Pie diagram (a) and Venn diagram (b) of metabolites after *Microcystis aeruginosa* exposed to erythromycin; Figure S3. PCA map (a) and PLS-DA score map (b) of metabolites after *Microcystis aeruginosa* exposed to erythromycin; Figure S4. Volcanic map of differential metabolites between different treatments; Figure S5. Clustering heatmap of top 50 differential metabolites ranked by relative abundance of *Microcystis aeruginosa* in B0 vs B1 (a), B0 vs B10 (b), B10 vs L10 (c), L0 vs L10 (d); Figure S6. Clustering heatmap of top 500 genes ranked by relative abundance of *Microcystis aeruginosa*; Figure S7. PCA map (a) and PCA 3Dscore map (b) between different treatments based on relative abundance of genes; Figure S8. Volcanic map of differentially expressed genes between different treatments; Figure S9. Schematic diagram of the differentially expressed KEGG Photosynthesis-antenna proteins pathway (map00196) in *Microcystis aeruginosa* under B0 vs B10 and B10 vs L10 comparisons, based on the KEGG database. Red and green boxes indicate upregulation and downregulation of gene expression, respectively; Figure S10. Sketch map of the differentially expressed KEGG Phosphonate and phosphinate metabolism pathway (map 00440) of *Microcystis aeruginosa* in the B10 vs L10 (modified from KEGG). The red box indicates upregulation of gene expression; Figure S11. Sketch map of the phosphate and amino acid transporters unit in the differentially expressed KEGG ABC transporters pathway (map 02010) of *Microcystis aeruginosa* in the B10 vs L10. The red box indicates upregulation of gene expression, the green box indicates downregulation of gene expression, and half a red box and half a green box indicate both upregulation and downregulation of gene expression. The pathway map is adapted from the KEGG database.
