# Supplementary material for: Metatranscriptomic and Metabolomic Insights Reveal Enhanced Colonial Microcystis aeruginosa Tolerance to Erythromycin in P-Limited Environment
Source: Toxics. 2026 Jul 27;14(8):660. doi: 10.3390/toxics14080660 (PMC13517662; doi:10.3390/toxics14080660)
Supplement: Supplementary file 1 [file toxics-14-00660-s001.zip › toxics-4422164-supplementary.pdf]

Supplementary Material for

Metatranscriptomic and metabolomic insights reveal enhanced colonial *Microcystis aeruginosa* tolerance to erythromycin in P-limited environment

Lei Jiang [a, b], Li-Jun Zhou [a, *], Shengxing Wang [a, c], Siwen Chen [d, e], Xiaoli Shi [a], Qinglong L. Wu [a, f, g, h], Kaining Chen [a, i *]

[a] State Key Laboratory of Lake and Watershed Science for Water Security, Nanjing Institute of Geography and Limnology, Chinese Academy of Sciences, Nanjing 211135, China

[b] University of Chinese Academy of Sciences, Beijing, 100049, China

[c] School of Life Sciences, Institute of Life Science and Green Development, Hebei University, Baoding 071002, China

[d] Key Laboratory of Aquatic Ecosystem Health in the Middle and Lower Reaches of Yangtze River, Ministry of Ecology and Environment, Jiangsu Provincial Academy of Environmental Science, Nanjing 210036, China

[e] Key Laboratory of Integrated Regulation and Resource Development on Shallow Lakes College of Environment Hohai University, Nanjing 210098, China

[f] Center for Evolution and Conservation Biology, Southern Marine Sciences and Engineering Guangdong Laboratory (Guangzhou), Guangzhou 511458, China

[g] Sino-Danish Center for Science and Education, University of Chinese Academy of Sciences, Beijing 100039, China

[h] The Fuxianhu Station of Plateau Deep Lake Research, Chinese Academy of Sciences, Yuxi 653100, China

[i] Collaborative Innovation Center of Technology and Material of Water Treatment, Suzhou University of Science and Technology, Suzhou 215000, China

Email address: ljzhou@niglas.ac.cn (Li-Jun Zhou)

* Corresponding author at: State Key Laboratory of Lake and Watershed Science for Water Security, Nanjing Institute of Geography and Limnology, Chinese Academy of Sciences, Nanjing 211135, China.

Email address: knchen@niglas.ac.cn (Kaining Chen)

* Corresponding author at: State Key Laboratory of Lake and Watershed Science for Water Security, Nanjing Institute of Geography and Limnology, Chinese Academy of Sciences, Nanjing 211135, China.

Contents of the Supplementary Material

Supplementary Texts

Text S1. Detailed extraction procedures and data analysis processes for metabolomic analysis

Text S2. Detailed experimental and analytical procedures for metatranscriptomic analysis

Supplementary Tables

Table S1. PERMANOVA analysis of metabolites at different treatments

Table S2. The numbers of differential metabolites

Supplementary Figures

Figure S1. The variation of the determined concentration of erythromycin after 12 days of culture at nominal concentrations of 1, 10, 20 and 100 μg/L, with a regular replenishment of erythromycin. Mean ± standard deviation of three replicates is shown for each value.

Figure S2. Pie diagram (a) and Venn diagram (b) of metabolites after *Microcystis aeruginosa* exposed to erythromycin.

Figure S3. PCA map (a) and PLS-DA score map (b) of metabolites after *Microcystis aeruginosa* exposed to erythromycin.

Figure S4. Volcanic map of differential metabolites between different treatments.

Figure S5. Clustering heatmap of top 50 differential metabolites ranked by relative abundance of *Microcystis aeruginosa* in B0 vs B1 (a), B0 vs B10 (b), B10 vs L10 (c), L0 vs L10 (d).

Figure S6. Clustering heatmap of top 500 genes ranked by relative abundance of *Microcystis aeruginosa*.

Figure S7. PCA map (a) and PCA 3Dscore map (b) between different treatments based on relative abundance of genes.

Figure S8. Volcanic map of differentially expressed genes between different treatments.

Figure S9. Schematic diagram of the differentially expressed KEGG Photosynthesis-antenna proteins pathway (map00196) in Microcystis aeruginosa under B0 vs B10 and B10 vs L10 comparisons, based on the KEGG database. Red and green boxes indicate upregulation and downregulation of gene expression, respectively.

Figure S10. Sketch map of the differentially expressed KEGG Phosphonate and phosphinate metabolism pathway (map 00440) of *Microcystis aeruginosa* in the B10 vs L10 (modified from KEGG). The red box indicates upregulation of gene expression.

Figure S11. Sketch map of the phosphate and amino acid transporters unit in the differentially expressed KEGG ABC transporters pathway (map 02010) of *Microcystis aeruginosa* in the B10 vs L10. The red box indicates upregulation of gene expression, the green box indicates downregulation of gene expression, and half a red box and half a green box indicate both upregulation and downregulation of gene expression. The pathway map is adapted from the KEGG database.

Text S1. Detailed extraction procedures and data analysis processes for metabolomic analysis

The extraction steps for the six groups (B0, B0.01, B1, B10, L0, and L10) were as follows: 1 mL of the sample was loaded onto an SPE column, and 3 mL of methanol eluate was collected. After drying under nitrogen, the residue was mixed well with 300 μL of pre-cooled solution (methanol/water, 4:1, v/v, containing 4 μg/mL internal standard). The mixture was ultrasonicated in an ice bath for 10 min, then incubated at -40°C for 2 h. Following centrifugation at 12,000 rpm (4°C, 10 min), 150 μL of the supernatant was filtered through a 0.22 μm membrane and transferred to an LC injection vial. Metabolite profiling was conducted using an ultra-performance liquid chromatograph (Waters, ACQUITY UPLC I-Class plus) linked to a high-resolution mass spectrometer (Thermo Fisher, QE HF) with a chromatographic column (ACQUITY UPLC HSS T3, 100 mm × 2.1 mm, 1.8 μm).

Raw data were preprocessed with the metabolomics software XCMS v4.5.1 for baseline correction, peak identification, integration, and retention time alignment. Compound identification was based on retention time (RT), exact mass, secondary fragment ions, and isotopic distribution, utilizing the Human Metabolome Database (HMDB), Lipidmaps (v2.3), METLIN, and the locally established LuMet-Animal 3.0 database by Oebiotech. The extracted data was subjected to RSD screening, missing value imputation, zero-value replacement, score-based filtering, log2 transformation, and dataset merging. After obtaining the metabolite data, differential metabolites

(DMs) were screened based on the criteria of p-value < 0.05 and Fold Change (FC) ≥ 1.2 or FC ≤ 1/1.2. Finally, the most significant biochemical metabolic pathways and signaling pathways associated with these DMs were identified through KEGG pathway enrichment analysis, with p-values calculated based on the hypergeometric test method.

Text S2. Detailed experimental and analytical procedures for metatranscriptomic analysis

A total of 2 mL of each sample was filtered through a 0.22 μm membrane prior to processing. rRNA was removed from qualified RNA using Illumina Ribo-Zero Plus rRNA Depletion Kits (Illumina, CA, USA) to enrich mRNA. The enriched mRNA was fragmented using fragmentation buffer, and first-strand cDNA was synthesized with random hexamers. Second-strand cDNA was generated by adding buffer, dNTPs, DNA polymerase I and RNase H, followed by purification with AMPure XP beads. The purified double-stranded cDNA underwent end repair, a-tailing, sequencing adapter ligation and size selection using AMPure XP beads. PCR amplification and subsequent bead purification were performed to construct the final library. Library quality was assessed by Qubit 2.0 quantification (diluted to 1 ng/μL), Agilent 2100 insert-size verification and q-PCR effective concentration determination (> 2 nM). Qualified libraries were pooled based on effective concentrations and target sequencing depth, loaded onto a flowcell for cluster generation via cBOT, and subjected to paired-end (PE) sequencing on the Illumina HiSeq platform. The bioinformatic analysis included

the following steps: Raw reads obtained from sequencing were first subjected to quality control to generate clean reads. Clean reads were then mapped to a pre-constructed metatranscriptomic unigene dataset. Gene abundance was calculated based on the number of reads mapped to each unigene. Finally, subsequent analyses integrated taxonomic and functional annotations of each gene from the metatranscriptomic project by comparing with the Kyoto Encyclopedia of Genes and Genomes (KEGG) database.

Table S1. PERMANOVA analysis of metabolites at different treatments

Compare	R^2	p	Significance
B0 vs B0.01	0.05757	0.87800	
B0 vs B1	0.18022	0.01731	*
B0 vs B10	0.43483	0.00667	**
B0 vs L0	0.40960	0.00667	**
B0 vs L10	0.44908	0.00954	**
B0.01 vs B1	0.17960	0.01731	*
B0.01 vs B10	0.42358	0.00667	**
B0.01 vs L0	0.42330	0.00667	**
B0.01 vs L10	0.45034	0.00667	**
B1 vs B10	0.40424	0.00667	**
B1 vs L0	0.53033	0.00900	**
B1 vs L10	0.54300	0.00667	**
B10 vs L0	0.54964	0.00667	**
B10 vs L10	0.51349	0.00667	**
L0 vs L10	0.17344	0.01929	*

Table S2. The numbers of differential metabolites

Compare	Up	Down	Sum
B0 vs B0.01	10	39	49
B0 vs B1	56	187	243
B0 vs B10	236	82	318
B1 vs B0.01	141	63	204
B1 vs B10	329	14	343
B10 vs B0.01	53	203	256
L0 vs B0	78	269	347
L10 vs B10	317	263	580
L10 vs L0	77	98	175

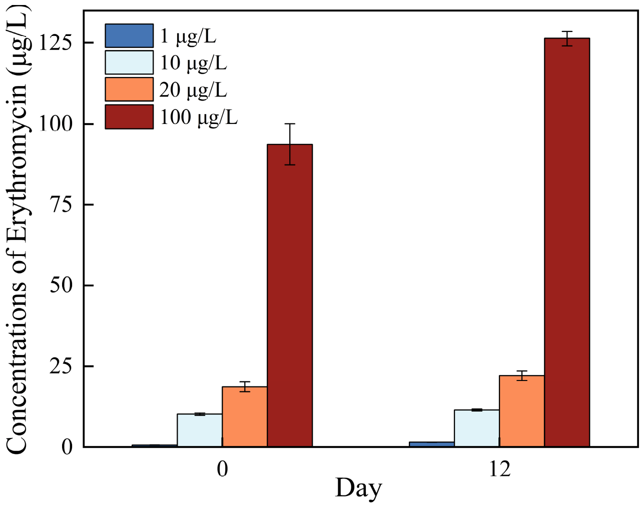

Figure S1. The variation of the determined concentration of erythromycin after 12 days of culture at nominal concentrations of 1, 10, 20 and 100 μg/L, with a regular replenishment of erythromycin. Mean ± standard deviation of three replicates is shown for each value.

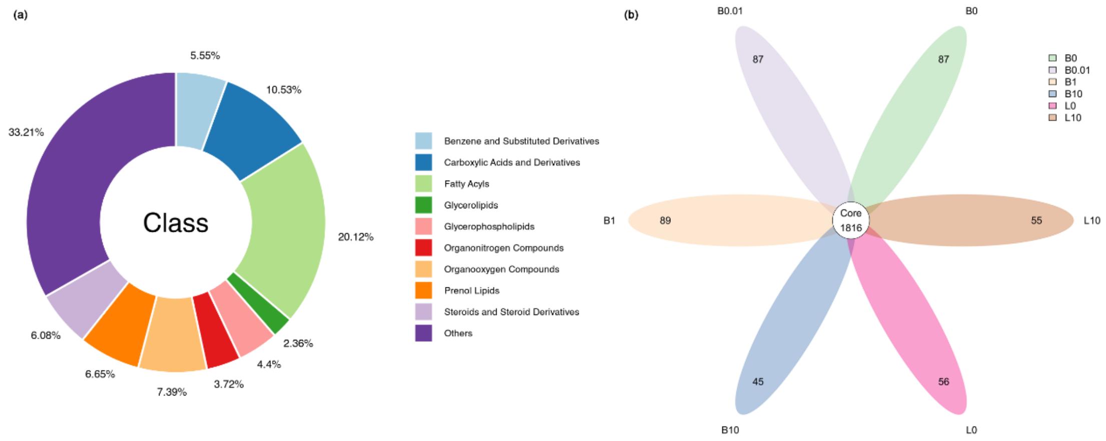

Figure S2. Pie diagram (a) and Venn diagram (b) of metabolites after *Microcystis aeruginosa* exposed to erythromycin.

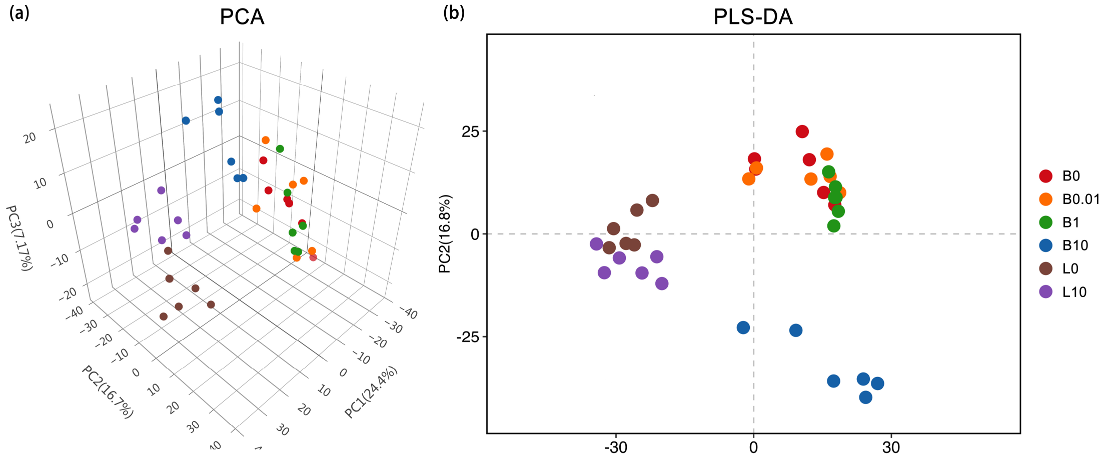

Figure S3. PCA map (a) and PLS-DA score map (b) of metabolites after *Microcystis aeruginosa* exposed to erythromycin.

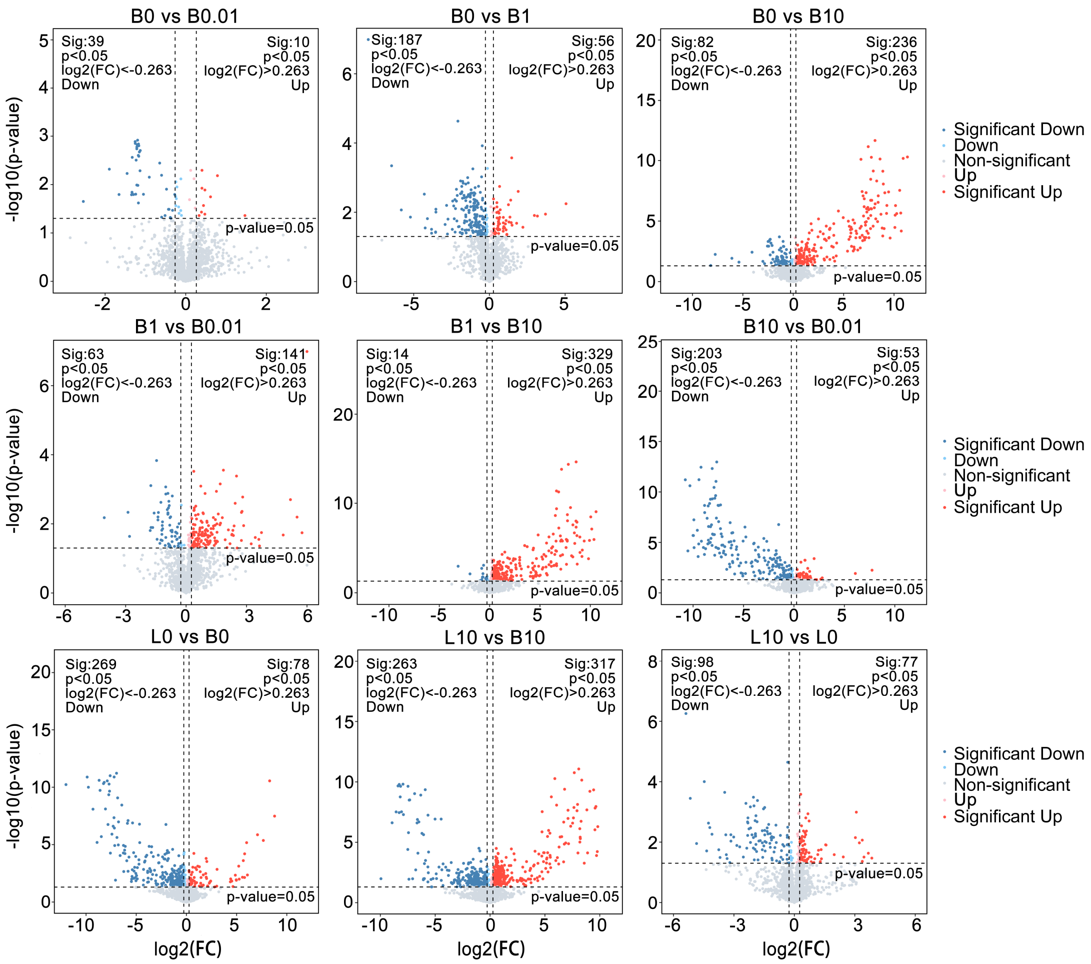

Figure S4. Volcanic map of differential metabolites between different treatments.

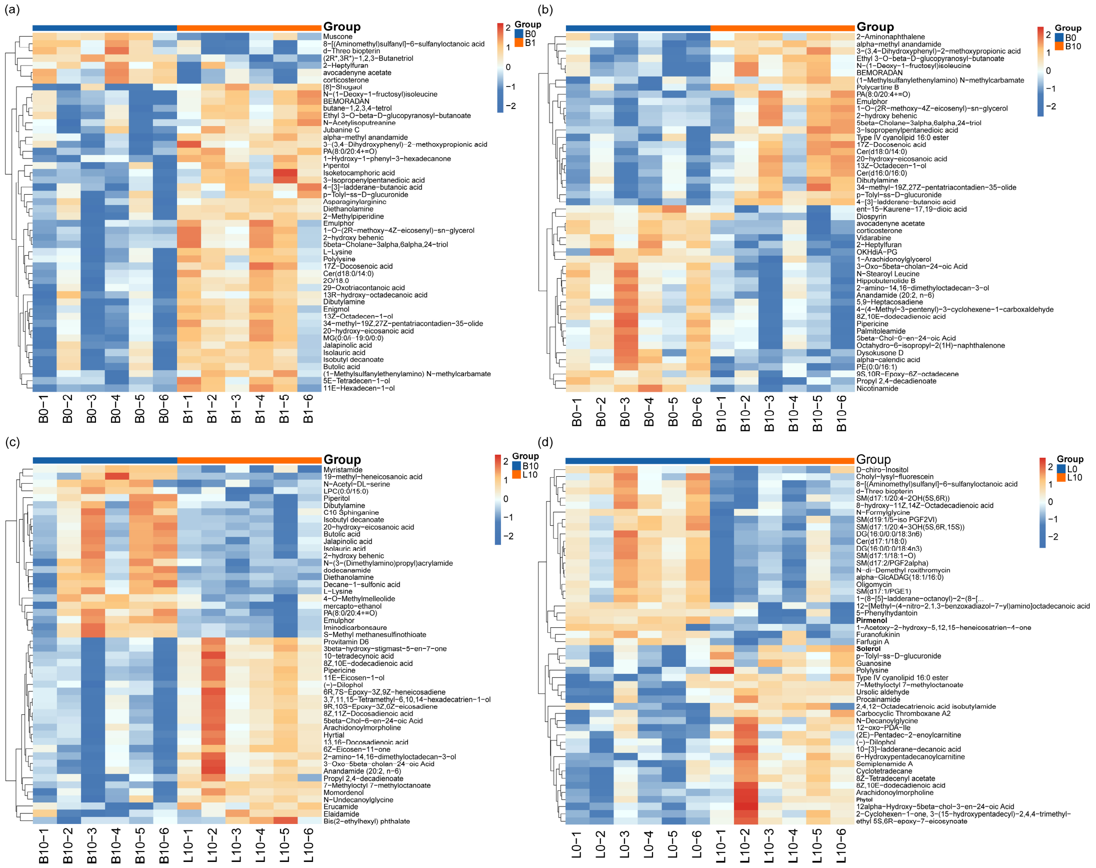

Figure S5. Clustering heatmap of top 50 differential metabolites ranked by relative abundance of *Microcystis aeruginosa* in B0 vs B1 (a), B0 vs B10 (b), B10 vs L10 (c), L0 vs L10 (d).

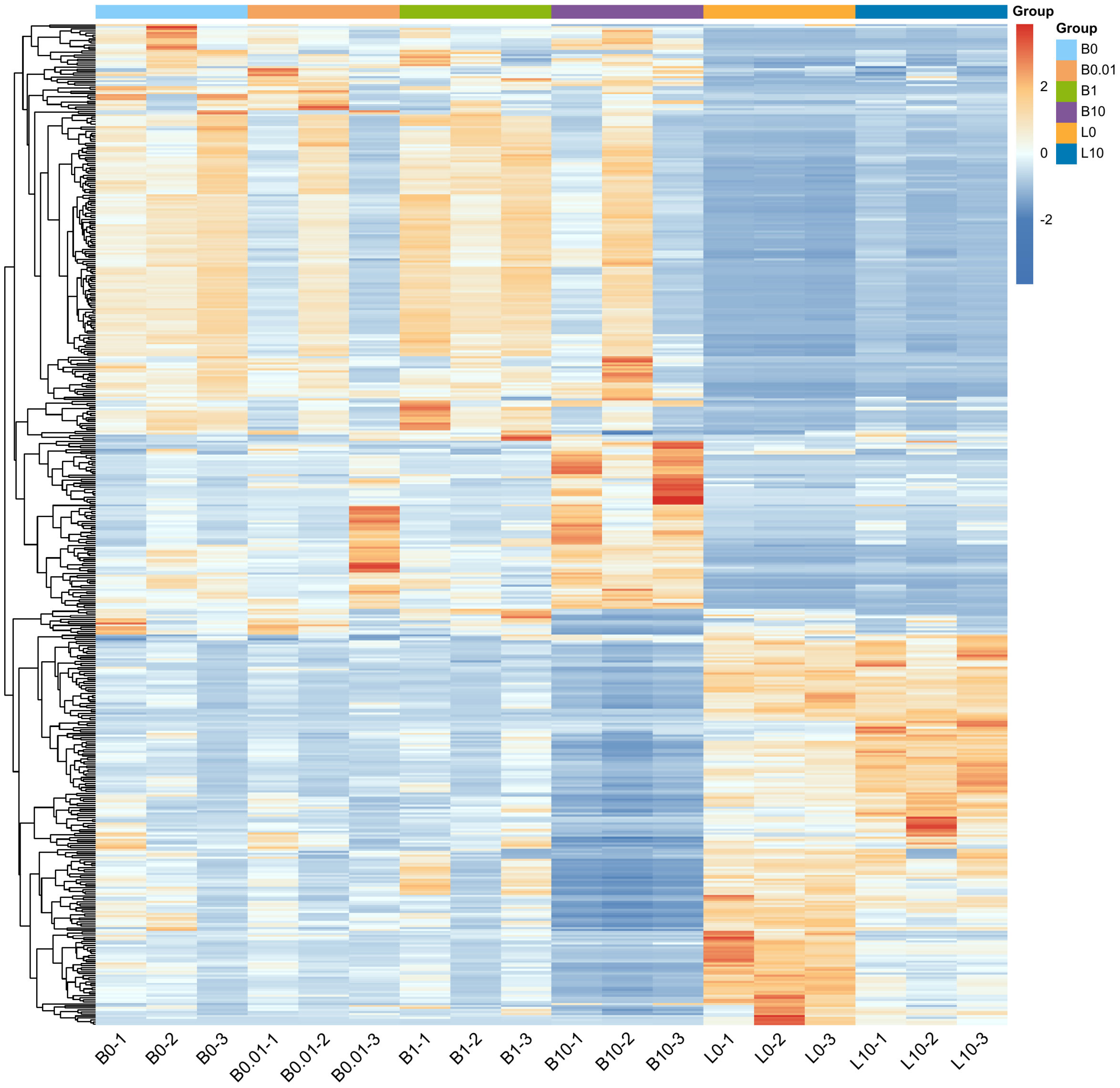

Figure S6. Clustering heatmap of top 500 genes ranked by relative abundance of *Microcystis aeruginosa*.

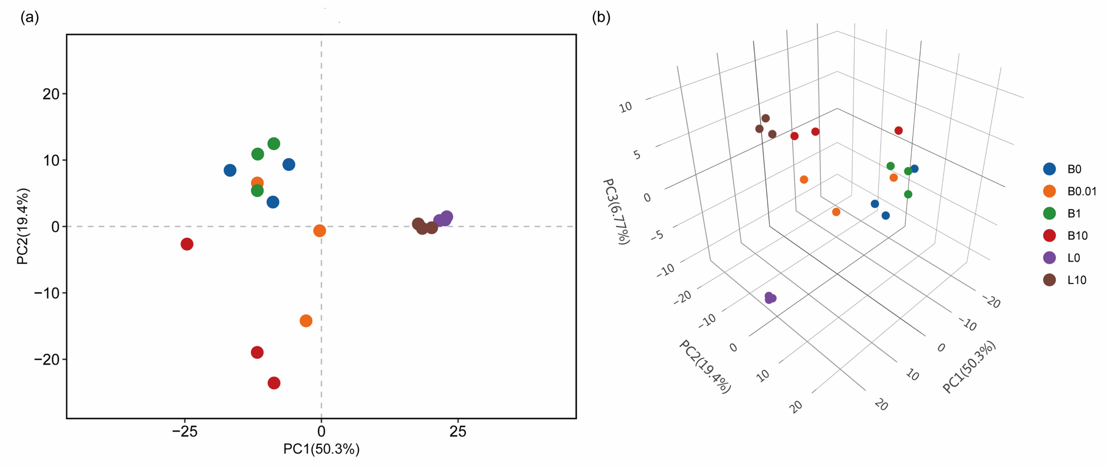

Figure S7. PCA map (a) and PCA 3Dscore map (b) between different treatments based on relative abundance of genes.

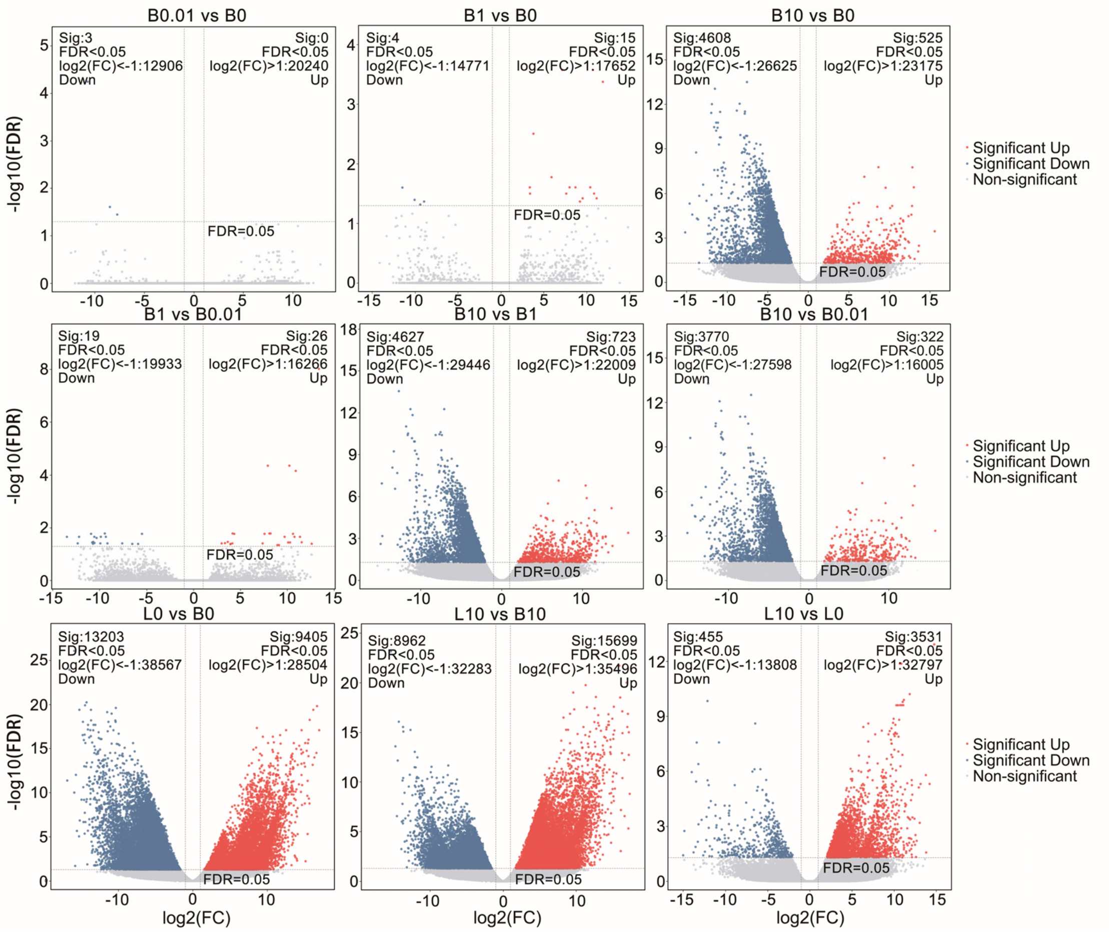

Figure S8. Volcanic map of differentially expressed genes between different treatments.

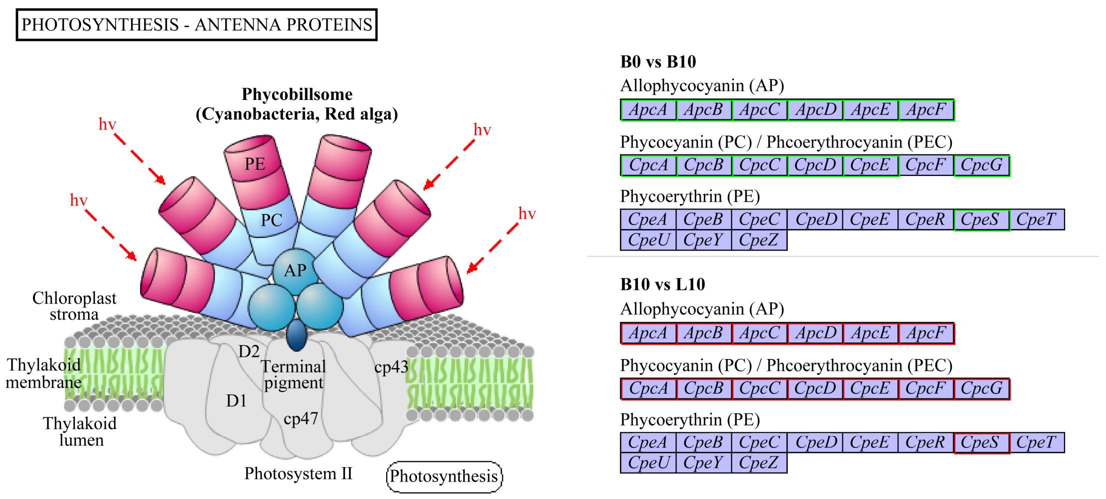

Figure S9. Schematic diagram of the differentially expressed KEGG Photosynthesis-antenna protein pathway (map00196) in *Microcystis aeruginosa* under B0 vs B10 and B10 vs L10 comparisons, based on the KEGG database. Red and green boxes indicate upregulation and downregulation of gene expression, respectively.

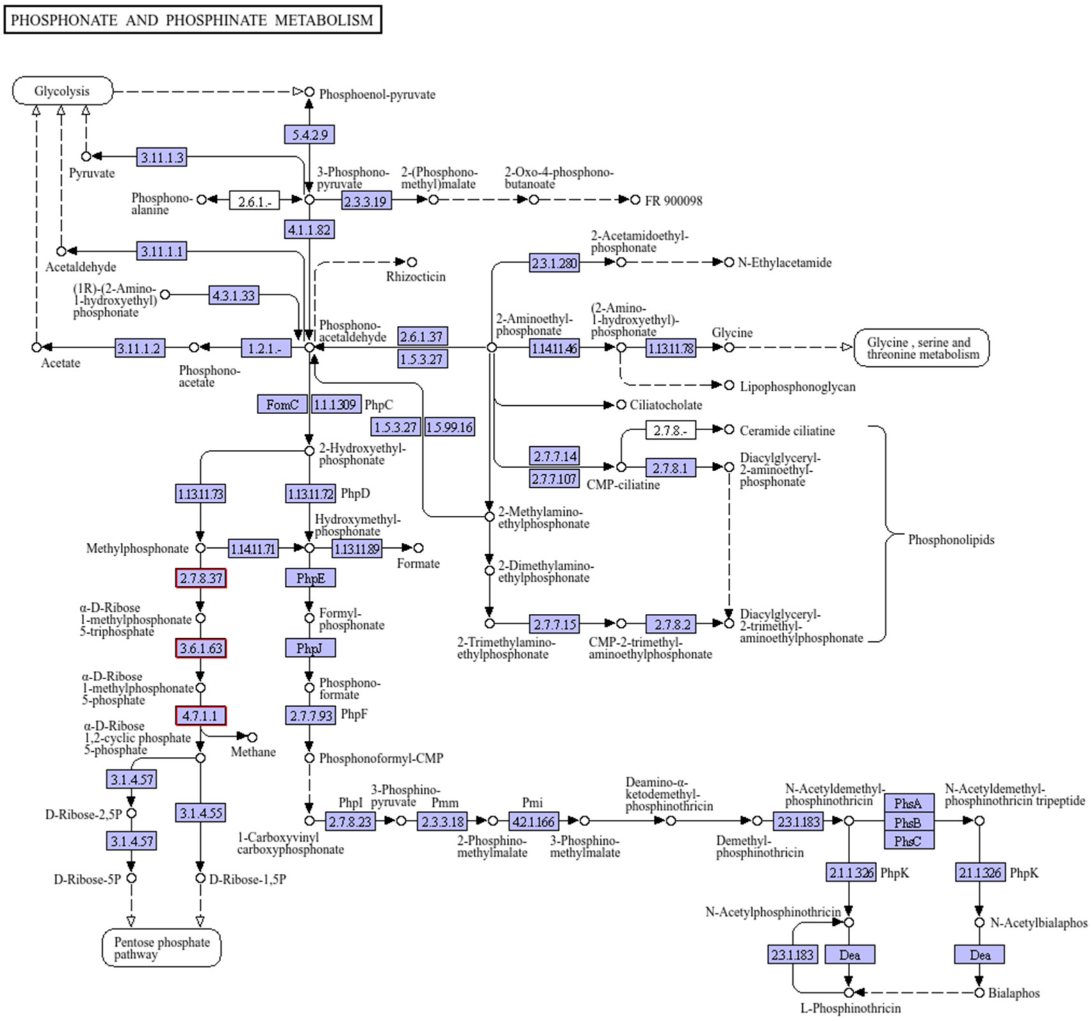

Figure S10. Sketch map of the differentially expressed KEGG Phosphonate and phosphinate metabolism pathway (map 00440) of *Microcystis aeruginosa* in the B10 vs L10 (modified from KEGG). The red box indicates upregulation of gene expression.

ABC TRANSPORTERS

Phosphate and amino acid transporters

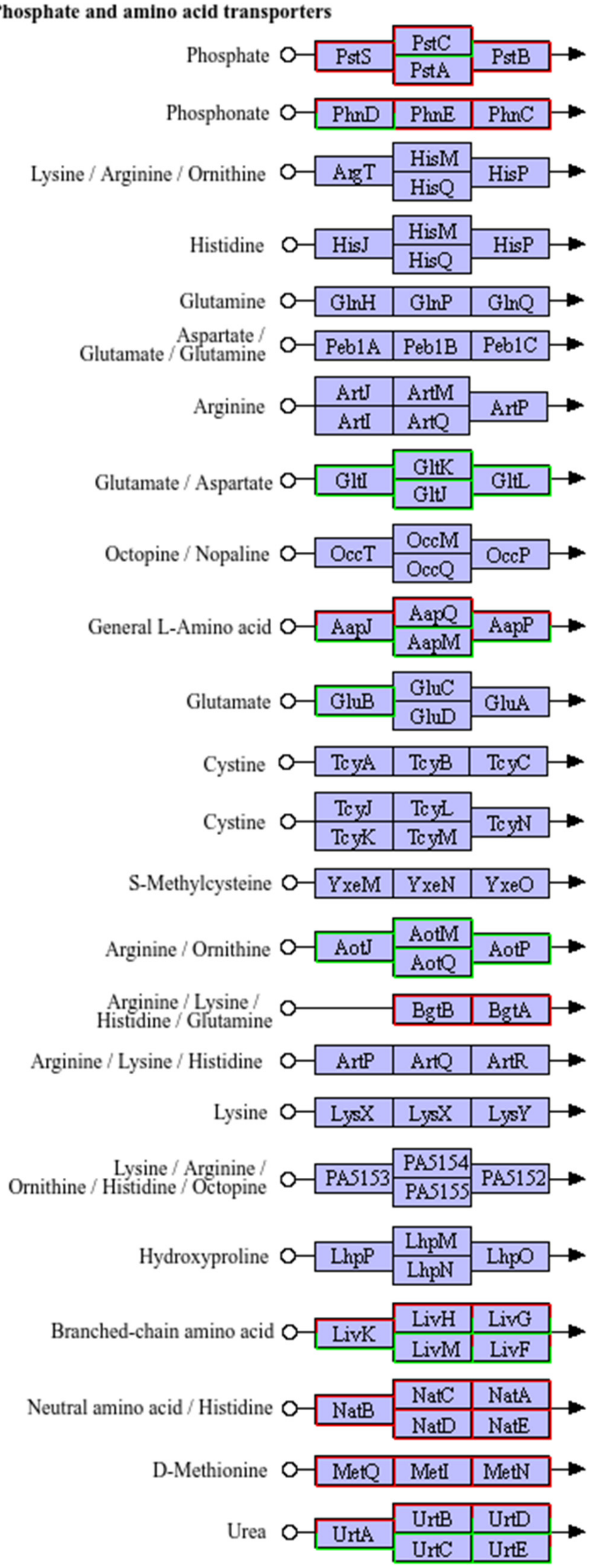

Figure S11. Sketch map of the phosphate and amino acid transporters unit in the differentially expressed KEGG ABC transporters pathway (map 02010) of *Microcystis aeruginosa* in the B10 vs L10. The red box indicates upregulation of gene expression, the green box indicates downregulation of gene expression, and half a red box and half a green box indicate both upregulation and downregulation of gene expression. The pathway map is adapted from the KEGG database.
